# Archives: The law of homologous series in variation (N. I. Vavilov)

**DOI:** 10.3897/CompCytogen.v14i3.54511

**Published:** 2020-07-14

**Authors:** Nina Bulatova

**Affiliations:** 1 A.N. Severtsov Institute of Ecology and Evolution, Moscow, Russia A.N. Severtsov Institute of Ecology and Evolution Moscow Russia

**Keywords:** genetics history, homologous variation, memory of Nikolai Vavilov, parallel evolution

## Editorial Preface

**Figure 1.**
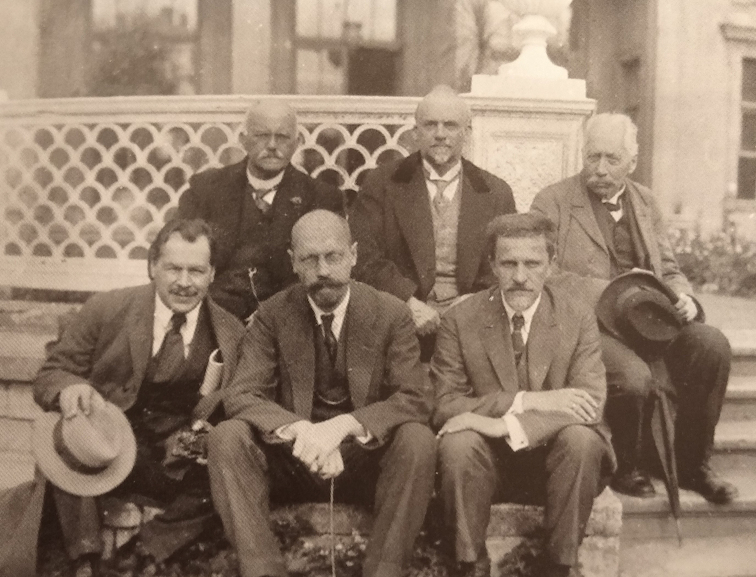
N. I. Vavilov (left below) and Russian geneticists V.A. Dogel, Yu.A. Filipchenko with the visiting European delegation: H. Federley, O. Fogt and W. Bateson (left to right in the second row). 1925, Leningrad (Vavilov 2012).

**Figure 2.**
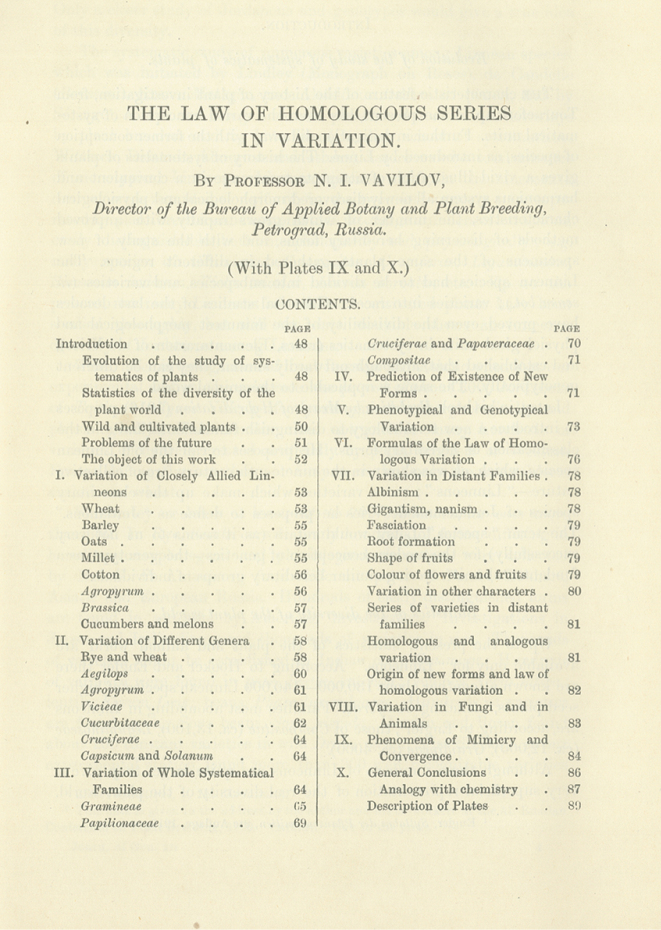
The title page of the Vavilov’s paper in the Journal of Genetics, 12(1), 1922.

A century has passed since the days when the law of homologous series in variation was first manifested. This event happened in 1920 in Saratov, in the third post-revolution year, in the frameworks of the III All-Russian Conference on Plant Breeding, then mobilized in view of current needs of agricultural practice, science and education. The report of a 33-year-old professor Nikolai Vavilov, who was accompanied by his students from the Saratov University, caused a sensation. Vavilov’s generalization on the phenomenon of the homologous series in variation of cultivated plants was reported on June, 4, 1920 and enthusiastically appreciated by the qualified breeders as a great scientific achievement comparable with the Mendeleev’s periodic Law of the chemical elements. On June 21, 1920, a message of the provincial Saratov branch of the Russian Telegraph Agency shared internationally the information on “the greatest discovery of world significance” which was addressed to the State government by the decision of the meeting. Very soon after the initial Russian publication (Vavilov 1920), the paper entitled “The Law of Homologous Series in Variation” was published in the Journal of Genetics, edited by W. Bateson and R.C. Punnett, the elder statesmen of genetics (Vavilov 1922). In 1925, William Bateson, Director of the John Innes Horticultural Institute, with colleagues, visited experimental fields and laboratories of Nikolai Vavilov, Director of the Bureau of Applied Botany and Plant Breeding (future N.I. Vavilov Institute of Plant Breeding) in a Petrograd – Leningrad (now Saint Petersburg) suburb (Fig. 1). The paper took 42 pages of Volume XII (1) (April, 1922, p. 47–89). The substance of this work by Vavilov was recalled in the chapters of N. Timofeeff-Ressovsky (1940) and N. Vavilov (1940) in the monograph “The New Systematics” (Huxley 1940), a synoptic book, preceding the publication on the new synthesis of theory of evolution (Huxley 1942). Since then and till now, genetic nature of homologous variation, the matter of the Vavilov’s law, has been in the focus of various disciplines, from agriculture to paleontology, being rejuvenated with the progress of molecular biology. Nowadays, molecular homology can be established universally at various levels, from unique genes to gene orders in chromosomes through genetic, cytogenetic and molecular analyses (Zakharov 1987) up to gene networks studied by bioinformatics (Suslov et al. 2008). It seems rational to meet the 100^th^ anniversary of this significant event of young hereditary science with a digital copy saving the author’s idea for future readers and investigators. The text is here reproduced in the Archives format from printed pages of the Introduction (p. 48–53) and the concluding section (p. 86–89) of the original English version (Vavilov 1922). The title page copied on Fig. 2 presents the whole contents of this work. Details of punctuation and citation are generally saved.

*N. Bulatova*

**Introduction [p. 48–53**]

*Evolution of the study of systematics of plants*

The characteristic feature of the history of plant investigation, from Tournefort up to the present, has been the varied conception of systematic units. Further investigation did away with the former conception of species, as introduced by Linné. The history of systematics of plants gives a vivid illustration of attempts to arrange in a convenient and harmonious system all newly discovered morphological and physiological characteristics, the number of which grows rapidly with improved methods of discerning hereditary forms, and with the study of new specimens of the same plants, gathered in different regions. The Linnean species had to be divided into subspecies and varieties (*in sensu bot*.); varieties into races. Genetical studies of the last decades have proved even the divisibility of the minutest morphological and physiological units in systematics (races, Elementararten of de Vries), and established that, although outwardly similar, they can be different genotypically. The same is applicable to the animal world.

Lotsy, in his book Evolution by Means of Hybridization (1916), proposes to introduce a new terminology to distinguish fundamental units in the classification of hereditary forms. He proposes to call the old Linnean species, which, as was shown in the nineteenth century, are of collective nature – “Linneons”; races, varieties, which make up the elementary species of Jordan and de Vries he proposes to define as “Jordanons”. The term “species”, Lotsy would retain (as it seems to us not very successfully) for the modern conception of genetics – the genotype, as a fundamental unit covering similar hereditary groups of individuals.

*Statistics of the diversity of the plant world*

Up to the present, statistics of the plant and animal world are available only for “Linneons”. According to Hooker and Engler there are known altogether about 130,000–140,000 Linnean species of higher seed plants, including *Coniferae*. Families most abounding in Linneons are, according to Engler^✩^, those of *Compositae* (ca. 13,100), *Leguminoseae* (ca. 12,000), *Gramineae* (ca. 4,000).

Although these numbers of Linneons are quite large, they give a very superficial representation of the real diversity of the plant world. Only a closer study of Jordanons and genotypes would give a true idea of this diversity.

The systematic study of numerous varieties among Linnean species, which was initiated by Lindley (Monograph on Roses), de Candolle (Brassica), Kraus, Metzger, and Alefeld on cultivated plants, and by Séringe^✩^, Jordan and Naegeli on wild plants, and is continued nowadays by plant breeders and by botanists (Swedish school of systematists: Wittrock, Dalstedt, Almquist and others), has revealed a total absence of monotypical Linneons. Linnean species, which, in the nineteenth century were regarded as uniform, in the twentieth century were separated by plant breeders and systematists into large numbers of Jordanons, easily distinguishable both morphologically and physiologically; e.g. many species of *Gramineae*, *Compositae*, *Cruciferae*, *Leguminoseae*, *Sesamum indicum*, *Viola tricolor*, *Linnea borealis*, etc. Up to the present, not many Linneons of wild and cultivated plants have been studied thoroughly, but still the data available shows an immense diversity of Jordanons among Linneons.

Thus, after investigations of local Russian and Asiatic wheats at our experimental station, the existence was proved of about 3000 Jordanons of *Triticum vulgare* Vill., perfectly recognizable morphologically and physiologically^✩^. This number does not include many hundreds of varieties of hybrids created artificially by plant breeders of Western Europe during the last thirty or forty years, but only the natural local varieties of wheat.

For barley we know at least 600 to 700 Jordanons, for oats more than 600. In Rye, *Secale cereale*, many hundreds of forms, differing in hereditary morphological and physiological characters, were collected by Mrs V. P. Antropova, from different parts of Persia, Bokhara, Asiatic and European Russia. Hundreds of easily distinguished forms are found in sorghum by American investigators. Investigations in Japan and India discovered thousands of varieties in rice. Thousands of varieties might be established in Indian corn, *Zea mays*. Hundreds of varieties were found in peas, *Pisum sativum*; vetches, *Vicia sativa*; lentils, *Ervum Lens*; beans, *Phaseolus vulgaris*. Hundreds of varieties are found among Soya beans, *Soya hispida*. Jordan and Rosen found about 200 constant varieties in wild *Draba verna*. Miss Sinskaja, at our experimental station, found more than 300 well recognizable varieties of *Eruca sativa*, a weed occurring in field of flax in Turkestan and Bokhara. Thousands of forms, perfectly distinguishable, exist among species of *Cucurbita Pepo*, *Cucurbita maxima*, *Citrullus vulgaris* – water-melon, *Cucumis sativus*, and *Cucumis Melo*^✩✩^. Hundreds of forms are found among wild *Linnea borealis* (Wittrock), *Picea excelsa* (Wittrock), etc.

*Wild and cultivated plants*

The majority of cultivated and wild Linneons propagated by seeds, are represented by hundreds of well-defined Jordanons. There is no essential difference in this respect between wild and cultivated plants. Wild Linneons, like clover (*Trifolium pratensis*), *Agropyrum cristatum*, *Agropyrum repens*, yellow alfalfa (*Medicago falcata*), *Alopecurus pratensis*, *Brassica elongata*, studied in detail at Russian Experimental Stations by plant breeders (Roudzinski, Lorch, Jegalov, Bogdan), proved to be no less variable than cultivated wheats, barleys, oats, and peas. The monotypic nature of many wild Linnean species is kept only so long as they are studied by a few specimens in the herbarium. The individual study in culture of many samples of the same Linneon inevitably discovers its polymorphic nature.

Still greater diversity is observable in plants multiplying vegetatively or apogamically, like roses, potatoes, apples, *Hieracium* (Naegeli), and *Dahlia*.

We do not exactly know if there are really monotypic Linnean species in nature, fairly well specific and separated from other Linnean species and represented by one variety, one Jordanon only. The whole impression is that the more we study our plants and animals, the more variable thay are, the more varieties we find among Linnean species. Several Linnean species of plants and animals, like roses, wheats, Indian corn, rice, squashes, *Drosophila*, seem to be extremely variable, but these have attracted more attention than others. We easily notice sharp differences in colour, size, and shape of several organs and are rather inattentive to others.

The differences of Jordanons within the limits of the same Linneon, in the shape and colour of their flowers, form and size of leaves, fruits and other organs, are very often no less marked than the differences between Linneons themselves. For instance, some varieties of *Cucurbita Pepo* are characterized by fruit the size of hen’s eggs; other varieties, growing under the same conditions, bear fruit three and four feet in diameter. Some varieties of *Sesamum indicum* have opposite leaves and fruits, others have alternate. Some varieties of wheat and rye have simple leaves, without differentiation into vaginae and plates, having no “ligula”, or “auriculae”; others have the usual complicated leaves, with “ligula”, and “auriculae”.

Plants self-fertilized, as wheat, barley, peas, soya, etc., and cross-fertilized, as rye, maize, beet, ale alike polymorphous. The seeming uniformity of several cross-fertilized wild and cultivated plants is only apparent when they are not studied carefully. The difference consists only in the homozygotic nature of many characters in cross-fertilized plants, and in the homozygotic nature of self-fertilized plants. Some recessive characters may be hidden in cross-fertilized plants by the dominance of other characters, but by artificial self-fertilization of these plants, and by inbreeding, it is possible to re-establish them. From what we know at present from the study of Indian corn (Emerson, Collins, and others), of rye, beetroot, *Drosophila*, man himself, cross-fertilized organisms are not less variable than self-fertilized.

The above mentioned numbers of Jordanons are in reality still greater, because, up to the present time, African and Asiatic varieties of even the most important cultivated plants, like wheat, oats, barley, peas, lentils, *Cruciferae*, are almost unknown.

*Problems of the future*

There is a real need for the study and systematizing of these Jordanons, especially in cultivated plants and domesticated animals, for the benefit of geneticists, as well as systematists and agriculturists. Only the closest study of Jordanons and genotypes will give a real re-presentation of what a Linneon is. To construct the general genetic schemes, it is necessary to know the composition of Linnean species. Before creating new varieties by crossing we ought to know what exists in nature. Even for cereals, *Leguminoseae*, and other most important plants, we have no adequate knowledge of even easily recognizable botanical varieties. Regions of ancient culture in Asia, Africa, and America still preserve numbers of varieties unknown to systematists and plant breeders.

In 1880, Alphonse de Candolle wrote in his remarkable book *La Phytographie*: “Un jour la science traitera les elements de l’espece comme les elements des genres, comme ceux de la famille et tous ces groupes seront coordonnes, les uns au-dessus des autres d’une maniere parfaitement uniforme” (p. 80). This day has arrived, but the task is not very simple. The closest study of some Linneons of cereals, *Leguminoseae*, *Cruciferae*, *Compositae*, and *Cucurbitaceae*, persuades one of the immensity of this work. The diversity of plants and animals is too great to admit of giving a complete list of existing forms. There comes the necessity to establish some principles and schemes of classification.

The near future promises to differentiate the Linneons still more, and to multiply the number of Jordanons and species in Lotsy’s sense. Artificial hybridization threatens considerably to enlarge the external diversity of forms.

It may be expedient to define even at the present time the multi-formity in Linneons, not by the number of described and possible compositions, but by the number and list of *varietal characters* through which Jordanons differ from each other, not forgetting that separate characters can be dependent on several hereditary factors or genes, involving complicated genotypical formulae. The complete genotypical compositions of Linneons is a problem for the future.

The multitudinous chaos of innumerable forms obliges investigators to look for some way of simplification. The process of differentiation will go on inevitably, adding to the records of existing forms, and giving a true conception of Linneons. But parallel to differentiation it is natural to search for ways of integration of our knowledge of Jordanons and Linneons themselves. If some 130,000 Linneons are difficult to manage for investigation, the work with tens and hundreds of millions of Jordanons will be still more complicated.

As formerly, in the study of dead organic and inorganic worlds, so at the present, the problem before the investigator of the animal and plant world is to explore the regularities in polymorphism, and to establish its classes.

*The object of this work*

Below is an attempt to integrate the phenomena of polymorphism which we define as “The Law of Homologous Series of Variation”. These regularities were noted by the author during the study of innumerable varieties of cultivated and wild plants.

The ideas expounded below in some parts are not foreign to biological literature. Separate facts of regular variation were known long ago. Naudin noticed them in his classical study of *Cucurbitaceae*. Darwin^✩^, who was in general rather the adherer of fortuitous variations in all directions in his Origin and Variation, paid attention to regular variation, which, as he states, “occasionally” happens in plants and animals.

M.J. Duval-Jouve collected a great many data on the variation of wild Linnean species of *Gramineae*, *Juncaceae* and *Cyperaceae* in his paper on “Variations paralleles des types congeneres” published in 1865 in *Bull. De la Ste. Botanique de France*, Vol. XII. His conclusions in some part come near to the statements of our study. De Vries notices in his *Mutationstheorie* the existence of series of variation^✩^. Eimer in his study of Orthogenesis approached the same subject from a different point of view. Several palaeontologists (Cope, Oscborn) noticed regular variation in animals. More recently Saccardo^✩✩^ and Zederbauer^✩✩✩^ gave extremely instructive instances of regular variation in fungi and *Coniferae*.

The detailed study of variation among many different groups, and the great number of new facts permits us to take this subject anew and bring all known facts into the form of a general law to which all organisms are submitted.

**X. General conclusions [p. 86–89**]

Parallelism in varietal polymorphism, and the existence of regularity in differentiation of greater groups as Linneons, genera, and families, is a great help in the study of varieties in self- and cross-fertilized plants and animals. Instead of searching for unknown forms, the investigator can definitely look for, and foresee, forms lacking in a system, by noticing the similarities with the nearest known Linneons and genera. In this respect a biologist places himself in the position of a chemist, who classifies substances according to their place in a system, and creates them through synthesis.

The investigation of polymorphism and the description of new forms become full of scientific meaning and interest. New forms have to fill vacancies in a system. The collections of immense numbers of butterflies and beetles in our museums and herbariums will play a more worthy role in the immediate future than ever before. For a systematist is not a man who knows all the curiosities of nature, but one who grasps the order and sense of it all.

The existing systems of Linneons and varieties ought to be fundamentally changed, and constructed according to a general plan. Instead of occasional characters, which usually determine species and varieties, it would be more rational to follow a general system. The greatest problem of systematists is to build up a general well sustained monotypical system, where similarity and homological series of variation would be considered as the fundamental basis, instead of an indefinite tangle of names impossible to remember. This may seem rather revolutionary for systematists, and it must be done very carefully, in consideration of existing orders. It would be easier to arrange in general systems of minutest systematical units, varieties and races which are as yet almost untouched by systematists. We have tried this for cultivated plants, and have found it expedient. Instead of remembering endless forms, usually named after occasional places of origin or in honour of persons, we have the possibility of studying a system and introducing into it individual additions, where it may be necessary to do so, for single Linneons and genera. We realize well the size and difficulty of the whole problem. Without a differential work, and without studying in detail, the integral work will be groundless. To integrate it is necessary to differentiate. We know that perhaps a century will pass before botanists and zoologists will create, through collective work, an organized world system; but this way is historically necessary and inevitable.

*Analogy with chemistry*

The above-mentioned analogy of the present day position of the biologist and chemist is deeper than it might seem at first. We have spoken conventionally about characters, colours, hairiness, beardedness, etc. Chemistry says little about the exterior of its substances; it considers the chemical nature of its compounds and their formulas. Numerous chemical substances are required to a harmonious system of combinations of a few elements. The biologist is still far behind. During the last decades, however, genetics has advanced greatly and is rapidly overtaking chemistry – at least the old chemistry of complicated organic compounds. Genetics is creating a laconic language of signs for hereditary factors, determining external characters. The biologist has learned to analyze organisms, and to get a hold on methods for the synthesis of new forms.

The regularities in polymorphism of plants, established by a minute examination of variation in different genera and families which we have examined, can be compared to homologous series of organic chemistry, e.g. carbohydrogen (CH_4_, C_2_H_4_, C_2_H_2_, …). Its series of compounds differing from each other, are still characterized by many common properties in reactions, by definite cycles of compounds, by definite reactions of exchange and adhesion. Every single hydrocarbon gives a series of compounds similar to that of other hydrocarbon.

In general, genera (*G*1, *G*2, *G*3, …) and Linneons (*L*1, *L*2, *L*3, …) of plants and animals display, in just the same manner, their homologous series of varieties, corresponding to different homologous series of hydrocarbons.

*G*1*L*1 (*a+b+c*…) _ _ _ _ *G*2*L*1 (*a+b+c*…)

*G*1*L*2 (*a+b+c*…) _ _ _ _ *G*2*L*2 (*a+b+c*…)

*G*1*L*3 (*a+b+c*…) _ _ _ _ *G*2*L*3 (*a+b+c*…)

*L*1*a*1, *L*1*a*2, *L*1*a*3, …

*L*2*a*1, *L*2*a*2, *L*2*a*3, …

*L*3*a*1, *L*3*a*2, *L*3*a*3, …

Where *a*1, *a*2, *a*3, … are different characters which distinguish different varieties. The series of forms are strikingly analogous to homologous series of organic chemistry.

Besides their chemical structure, different forms of organized nature are characterized by physical structure, and perhaps it would be better to trace also the analogy of homologous series of plants and animals, with systems and classes of crystallography with definite chemical structure (Crystallo-Chemistry of Fedoroff).

We leave the question, in detail, of these analogies, which is already discussed in literature (Johannsen, Lohmann, Tischler). Further investigations will establish more precisely the law of homologous variation in plants and animals, and it may be posiible to bring the same series into mathematical expression. The variation in form might be reduced to some geometrical scheme.

The problem of the origin of species cannot be separated from the problem of variation. A great many forms are undoubtedly only different combinations of the same genes, some primary types. The study of variation will give us the possibility of establishing these primary types, the fundamental series of variation of organisms.

The idea of the homologous series in variation in its essence is only a development of the general idea of Goethe’s “Metamorphosis of plants”, the idea of the unity in variety of C. Dresser^✩^.

In conclusion, we take the liberty of expressing our strong conviction that the most rational and expedient method of studying the diversity of plants and animals open to breeders of both, even for practical purposes, is through the establishment of parallelism and homologous series of variation.

## Footnotes

Engler, Syllabus der Pflanzenfamilien, 8te Auflage, 1919.

Musee helvetique, p. 115 (Aconitum).

This data is given in the address by the author and his co-workers at the All-Russian Conference on Plant Breeding, 1920. Saratov. Now in the press.

These plants were studied at our experimental station by Mr S.M. Boukasov and Mrs S.A. Kartashov.

Darwin, Variation of Animals and Plants, Part 2; “Analogous or Parallel Variation.”

G.H.T. Eimer, Die Entstellung der Arten auf Grund von erworbener Eigenschaften nach den Gesetzen organischen Wachsens, Vols. I-III. 1888–1901, Jena.

P.A. Saccardo, “I Prevedibilli Funghi Futuri secondo la Legge d’Analogia”. Degli Atti dei R. Instituto Veneti de Scienze, Lettere ed Arti, Tome VIII. Ser. 7.

E. Zederbauer, “Variationsrichtungen der Nadelholzer”. Sitzberichte d. Akademie d. Wissenschaften, Wien, Math. Nat. Klasse, 116, Abt. 1. 1907.

Christofer Dresser, Unity in Variety. London, 1860. Recently there appeared several works devoted to the general uniformity of phenomena of life, history, psychology. See f.i. K. Marbe, Die Gleichformigkeit in der Welt. Bd. I and II. Munchen 1916–1919. P. Kammerer, Das Gesetz der Serie, 1919.
